# Implications of Anomalous Pectoralis Muscle in Reconstructive Breast Surgery: The Oblique Pectoralis Anterior

**Published:** 2012-09-10

**Authors:** Katherine Marie Huber, Travis Guthrie Boyd, Amy R Quillo, Bradon J Wilhelmi

**Affiliations:** ^a^School of Medicine; ^b^Department of Surgery, Division of Surgical Oncology; ^c^Department of Surgery, Division of Plastic and Reconstructive Surgery, University of Louisville, Louisville, Ky

## Abstract

**Introduction:** Many case reports have described anatomical variants of the pectoralis muscles. However, there is a paucity of published literature on the consequence of such presentations in reconstructive breast surgery. **Methods:** A 45-year-old female patient with breast cancer presented for left mastectomy and immediate reconstruction with tissue expander. During mastectomy, she was noted to have an extra muscle anterior to her pectoralis major muscle. This variant had not previously been described in the literature and was therefore named the oblique pectoralis anterior. After inspection of the aberrant musculature, the decision was made to release the inferolateral insertion of the accessory muscle with the inferior edge of pectoralis major. An adequate pocket for the expander was created. **Results:** After routine expansion and implant exchange, muscular coverage of the implant from pectoralis major and the oblique pectoralis anterior muscle approximated 70%. The patient was left with good symmetry and a cosmetic result, despite the challenges presented by her anomalous chest wall musculature. **Discussion:** Prior knowledge of the various anatomic aberrations described in the literature can prepare a surgeon to properly incorporate and utilize the variant anatomy, should it be encountered, to benefit the outcome of the operation.

A plethora of anatomical variations of the pectoralis major muscle have been described in the literature. None appear to have a functional role and they are often described as vestiges or developmental aberrations. Many case studies are published, but the infrequency of these anomalies has made broad anatomical studies impractical. There are no reliable clinical tests for the presence of abnormal pectoralis tissue when not visibly obvious, and muscular variants are rarely noted on diagnostic imaging before open operations.^1^ It is therefore important for the surgeon to be aware of anomalous pectoralis major musculature and the clinical significance of such presentations. The authors present what they believe to be a unique case of an anomalous slip of pectoralis major, the oblique pectoralis anterior, and utilize it and the published literature to discuss the implications of anomalous musculature in breast reconstruction.

## METHODS

**Figure 1:**
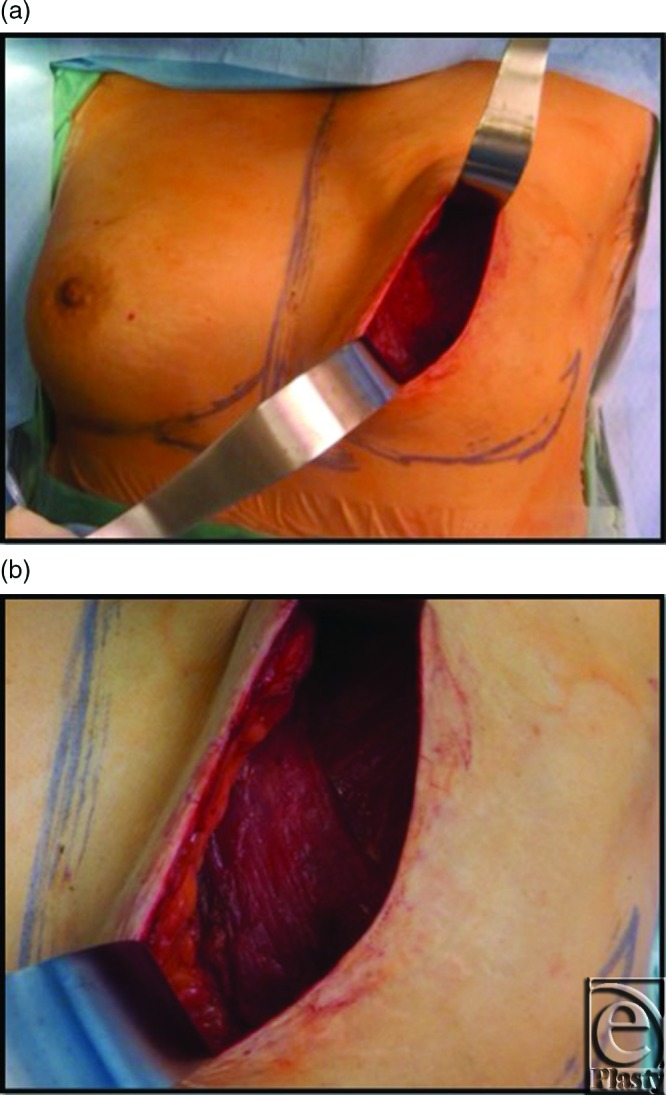
This intraoperative photograph of the left breast (*a*) demonstrates the aberrant pectoralis accessory muscle, the oblique pectoralis anterior, with its fibers perpendicular (*b*) to the underlying pectorals major muscle.

**Figure 2:**
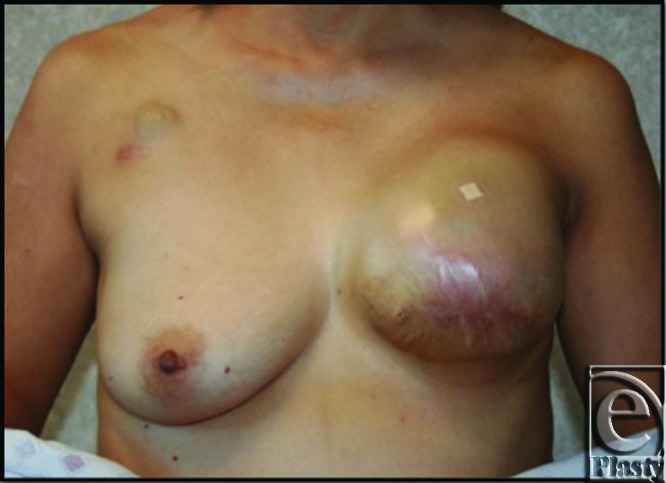
This photograph demonstrates the patient after her last expansion prior to the expander to implant exchange. Notice the small bandage over the port.

**Figure 3:**
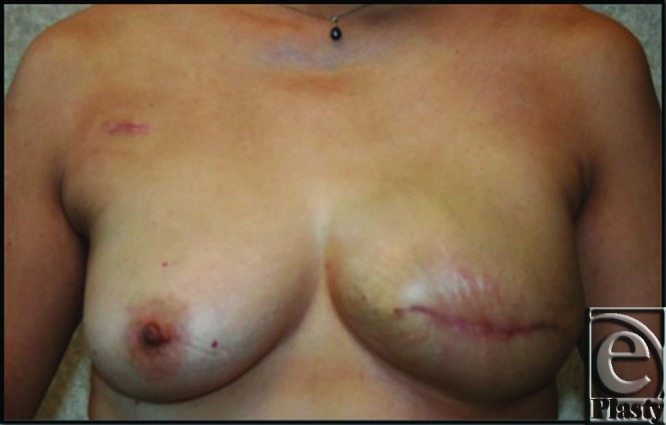
This photograph demonstrates the patient after expander to implant exchange. The permanent implant was placed under both the oblique pectoralis anterior and pectoralis major muscles. A 600 cc high-profile mentor memory gel implant was used for her reconstruction.

This 45-year-old patient underwent left mastectomy for breast cancer and immediate reconstruction with tissue expander. At the time of her mastectomy, she was noted to have an extra muscle anterior to her pectoralis major muscle. The fibers of this extra muscle were oriented obliquely and in the perpendicular direction to the underlying pectoralis muscle (see Fig 1). This oblique pectoralis anterior muscle originated from the sternum medially and coursed inferiorly to insert on ribs 5 and 6 and the anterior rectus aponeurosis. The pectoralis major muscle was normally developed underneath this accessory pectorals muscle. During the dissection to create the pocket for the insertion of the expander, the oblique pectoralis anterior muscle was released inferiorly along the same level as the pectoralis major muscle. The subpectoral pocket was created, preserving the pectoralis minor muscle. Specifically, a mentor CPX3 350 cc tall profile expander was used for her expansion. Her expansion course was routine and lasted 4 months until the distance from the clavicle to inframammary fold matched her right breast and chest wall, which was 29 cm. The total amount of left expander fill at this point was 540 cc. (see Fig 2). Moreover, her left mastectomy specimen weight was 535 g. Once this goal measurement of 29 cm was met, we waited 1 more month to minimize contraction at implant exchange. At her expander exchange operation, the expander was deflated and removed through her previous incision. Superior, inferior, and medial capsulectomy was required to adjust her subpectoral pocket to accommodate the 600 cc high-profile mentor memory gel implant. (see Fig 3) No acellular dermal matrix was utilized in this implant procedure.

## RESULTS

Muscular coverage of the implant from pectoralis major and the oblique pectoralis anterior muscle approximated 70%. The remaining lower pole of the implant was covered by soft tissue, as in any other submuscular implant. The patient was left with a satisfactorily cosmetic result, despite the challenges presented by her anomalous chest wall musculature.

## DISCUSSION

When discussing muscular variants and their significance, it is prudent to first be familiar with the normal anatomy of pectoralis major. The clavicular head of the muscle attaches superiorly to the medial two thirds of the clavicle, while the sternocostal head originates medially on the lateral edge of the sternum and inferiorly from the aponeurosis of the external oblique at the level of the sixth and seventh ribs. The muscle inserts laterally in the intertubercular groove of the humerus. The pectoralis major muscle is a prime flexor, adductor, and medial rotator of the humerus. Innervation is by the medial and lateral pectoral nerves.^2^

Familiarity with the anatomic variations of pectoral musculature is important, as is identifying them early to achieve appropriate dissection planes during surgery of the chest wall.^3^ Variations demonstrated in the literature are numerous. A commonly noted anomaly of this muscle group is its complete deficiency. Absence of pectoralis major has been described extensively, both as an isolated anomaly, and in association with congenital syndromes. Poland syndrome was first described in the literature in 1962 as a condition characterized by unilateral absence of pectoralis major and cutaneous syndactyly of the ipsilateral hand, although the first noted case dates back to 1841.^4^ Cilingir et al^5^ depict a case of unilateral pectoralis muscle agenesis with associated ipsilateral microtia (malformation of the ear). Spear et al^6^ describe a handful of patients diagnosed with Poland syndrome who, upon closer inspection, had completely normal pectoralis musculature and simply suffered from breast malformation, a phenomenon they described as anterior thoracic hypoplasia.

Beyond its complete absence, varying levels of hypoplasia of pectoralis major are described. Paraskevas et al present a case of bilateral hypoplasia of the clavicular head, with associated vascular anomaly in an anteriorly placed external jugular vein. They refer to this presentation as “atypical Poland syndrome.”^7^ Similarly, Soni et al elucidate a prominent cleft separating sternocostal and clavicular heads of pectoralis major unilaterally in which the cephalic vein traversed superficial to the clavicular portion. In this particular case, the deltopectoral groove was obliterated by the abnormal musculature.^3^ Unilateral subtotal aplasia of pectoralis major was described by Denti et al, which was associated with anomalous insertion. The fibrous “neotendon” that replaced the normal musculature in 2 separate cases coursed from the sternum to the medial humoral condyle covered by a thin layer of skin.^8^

Various accessory slips of muscle have been noted, some of which are named (see Table 1). A slip arising from the anterior border of latissimus dorsi and inserting into the biceps fascia is christened “axillary arch of Langer.” One arising from pectoralis major itself and inserting into the same biceps fascia is called “chondrohumeralis.” These 2 variants coexisted in a patient and have been described together by Lama et al, as both traverse superficial to the axillary neurovascular bundle, and can present with neurovascular complications.^9^ A slip arising deep to pectoralis major and superomedial to pectoralis minor is called pectoralis minimus. This variant is notable because the thoracoacromial vessels pass between it and pectoralis minor, and patients can have vascular symptoms with hyperextension of the arm.^10^ Arican et al presented a case of co-existing named anomalous slips. Pectoralis quartus arises from the costochondral junction of fifth and sixth rib and extends as a long flat band under the border of pectoralis major to insert into the intertubercular groove of the humerus. Another slip, pectoralis intermedius, arises from the third and fourth ribs, travels between pectoralis minor and pectoralis quartus, and merges with the tendon of the short head of biceps brachii.^11^ Finally, Loukas et al describe an unnamed variant originating from the border of serratus anterior, inferior to the abdominal head of pectoralis major, with normal insertion.^12^ Perhaps the most widely described variant is the “sternalis” muscle, which runs vertically, parallel and lateral to the sternum, anterior to pectoralis major. In one case, it is described as a “ribbon-like strap” from the costal region to the upper sternum over the medial border of pectoralis major.^13^ Some authors find it to be a variation of pectoralis major when innervated by pectoral nerves, while others find that because it is often innervated by intercostal nerves, it is an aberrant abdominal muscle.^11^^,^^14^ One group of authors describes another variation of the sternalis muscle arising unilaterally from the aponeurosis of the external oblique, dividing into a “Y” shape and then merging with sternocleidomastoid. This variant is aptly named “rectus thoracis bifurcalis.”^15^ Systemic anomalies have been connected to the presence of a sternalis muscle. Included are anomalies of the skull and adrenal gland, and other variations in pectoralis major muscles, most of which are described earlier. Occurrence of sternalis muscle in patients with anencephaly is also increased.^1^

Breast reconstruction with implants can be challenging for unilateral defects, especially in the setting of anomalous musculature. Symmetry is often paramount in creating a favorable cosmetic outcome and achieving patient satisfaction. The goals for expansion are for the expander to have stretched the skin a similar distance from the inframammary fold to the clavicle on each side. Ideally, the reconstructed breast base should also match the contralateral breast in the horizontal axis. This is of value in selecting the shape and size of the permanent implant. In breast reconstruction after mastectomy, high-profile implants, such as the high-profile mentor memory gel implant, are preferred, but measuring and comparing the existing breast projection and diameter can further refine implant selection. It is widely accepted that implant-based breast reconstruction is performed by placing the implant in a pocket created underneath the pectoralis major muscle. However, the presence of aberrant muscle slips can affect the size and origin of pectoralis major. Schulman et al note that in the presence of a sternalis muscle, pectoralis major often does not originate the edge of the sternum but instead originates lateral to the aberrant muscle. Lifting a sternalis muscle with pectoralis major helps to avoid a small, laterally displaced pocket for the expander.^1^ The patient in our case was found to have a distinct aberrant muscle anterior to the pectoralis major that also could have altered the ultimate cosmetic result. The authors had not previously encountered this variant in practice, nor subsequently did they encounter it in a review of the literature, as noted earlier. It has therefore been named the oblique pectoralis anterior. The perpendicular nature of the muscle was such that if it were not raised with the pectoralis major, it could have displaced the pocket, prolonged the expansion process, or caused additional scarring, ultimately leading to dissymmetry. For this reason, at the time of the reconstruction, the oblique pectoralis anterior muscle was released inferiorly in continuity with the pectoralis major muscle to maintain blood supply from perforators. The aberrant anatomy provided for additional muscle coverage and protection over the majority of the expander and, ultimately, the permanent implant. This patient went on to have excellent symmetry and no rippling or wrinkling. (see Fig 3). Prior knowledge of the anatomic aberrations described earlier can prepare a surgeon to properly incorporate and utilize the variant anatomy, should it be encountered, to benefit the outcome of the operation. In this case, the newly described pectoralis variant, the oblique pectoralis anterior muscle, was safely incorporated in this breast reconstruction for placement of subpectoral expander and permanent implant with a favorable outcome.

## Figures and Tables

**Table 1:** Anatomical descriptions of anomalous pectoralis musculature reported in the literature

Name	Origin	Insertion	Description	Reference
**Chondrohumeralis**	Normal, part of pectoralis major medially	Superficial to axillary neurovascular bundle into fascia of biceps	Muscular slip	9
**Axillary arch of Langer**	Anterior border of latissimus dorsi	Superficial to axillary neurovascular bundle into fascia of biceps, or into pectoralis major	Can be a single band or multiple bands	
**Sternalis**	From the inferior costal region, often from the aponeurosis of the external oblique	The infraclavicular region, often on the sternal tendon of the sternocleidomastoid	Also called anomalous sterni, parasternal, rectus sternalis, etc… Fibers run vertically parallel to the sternum	1,13
**Rectus thoracis bifurcalis**	External oblique aponeurosis	Merges with sternocleidomastoid bilaterally	Splits into “Y” shape at sternal angle	15
**Pectoralis minimus**	Second costal cartilage	Superior surface of the coracoid process	Deep to pectoralis major and superomedial to pectoralis minor, becomes tendinous laterally	10
**Pectoralis quartus**	Costochondral junction of the fifth and sixth ribs	Intertubercular groove of the humerus	Extends laterally under the border of pectoralis major	11
**Pectoralis intermedius**	Third and fourth ribs	United with tendon of short head of biceps brachii	Fleshy slip between pectoralis minor and pectoralis quartus	
**Anomalous pectoralis major**	Inferiorly from serratus anterior	Intertubercular groove of the humerus	An accessory head of pectoralis major arising inferiorly	12

## References

[1] Schulman MR, Chun JK (2005). The conjoined sternalis-pectoralis muscle flap in immediate tissue expander reconstruction after mastectomy. Ann Plast Surg.

[2] Morton DA, Foreman KB, Albertine KH, Morton DA, Foreman KB, Albertine KH (2011). Anterior thoracic wall. The Big Picture: Gross Anatomy.

[3] Soni S, Rath G, Suri R, Kumar H (2008). Anomalous pectoral musculature. Anat Sci Int.

[4] Clarkson P (1962). Poland's syndactyly. Guys Hosp Rep.

[5] Çilingir M, Malkoç C, Duman A, Eroglu S, Karacaoglan N (2004). Microtia and pectoralis muscle agenesis. Plast Recon Surg.

[6] Spear SL, Pelletiere CV, Lee ES, Grotting JC (2004). Anterior thoracic hypoplasia: a separate entity from Poland Syndrome. Plast Recon Surg.

[7] Paraskevas GK, Raikos A (2010). Bilateral pectoral musculature malformations with concomitant vascular anomaly. Folia Morphol.

[8] Denti M, Facchini R, Peretti G (1985). Partial aplasia of the pectoralis major muscle with an anomalous distal insertion: two case reports. J Pediatric Ortho.

[9] Lama P, Potu BK, Bhat KM (2010). Chondrohumeralis and axillary arch of Langer: a rare combination of variant muscles with unique insertion. Rom J Morphol Embryol.

[10] Rai R, Ranade AV, Prabhu LV, Prakash, Rajanigandha V, Nayak SR (2008). Unilateral pectoralis minimus muscle: a case report. Int J Morphol.

[11] Arican RY, Coskun N, Sarikcioglu L, Sindel M, Oguz N (2006). Co-existence of the pectoralis quartus & pectoralis intermedius muscles. Morphologie.

[12] Loukas M, South G, Louis RG, Fogg QA, Davis T (2006). A case of an anomalous pectoralis major muscle. Folia Morphol.

[13] Pinhal-Enfield G, Varricchio P, DeFouw DO, Vasan NS (2011). Sternalis muscle: importance of its awareness in chest imaging and clinical significance. Int J Anat Variations.

[14] O'Neil MN, Folan-Curran J (1998). Case report: bilateral sternalis muscles with a bilateral pectoralis major anomaly. J Anat.

[15] Mehta V (2010). Rectus thoracis bifurcalis: a new variant in the anterior chest wall musculature. Rom J Morphol Embryol.

